# A Road Map for Remote Digital Health Technology for Motor Neuron Disease

**DOI:** 10.2196/28766

**Published:** 2021-09-22

**Authors:** Ruben P A van Eijk, Anita Beelen, Esther T Kruitwagen, Deirdre Murray, Ratko Radakovic, Esther Hobson, Liam Knox, Jochem Helleman, Tom Burke, Miguel Ángel Rubio Pérez, Evy Reviers, Angela Genge, Frederik J Steyn, Shyuan Ngo, John Eaglesham, Kit C B Roes, Leonard H van den Berg, Orla Hardiman, Christopher J McDermott

**Affiliations:** 1 UMC Utrecht Brain Centre University Medical Centre Utrecht Utrecht Netherlands; 2 Biostatistics & Research Support Julius Centre for Health Sciences and Primary Care University Medical Centre Utrecht Utrecht Netherlands; 3 Department of Rehabilitation University Medical Centre Utrecht Utrecht Netherlands; 4 Center of Excellence for Rehabilitation Medicine University Medical Centre Utrecht and De Hoogstraat Rehabilitation Utrecht Netherlands; 5 Academic Unit of Neurology Trinity College Dublin Dublin Ireland; 6 Department of Physiotherapy Beaumont Hospital Dublin Ireland; 7 Faculty of Medicine and Health Sciences University of East Anglia Norwich United Kingdom; 8 Euan MacDonald Centre for Motor Neuron Disease Research University of Edinburgh Edinburgh United Kingdom; 9 Norfolk and Norwich University Hospital Norwich United Kingdom; 10 Alzheimer Scotland Dementia Research Centre University of Edinburgh Edinburgh United Kingdom; 11 Centre for Cognitive Ageing and Cognitive Epidemiology University of Edinburgh Edinburgh United Kingdom; 12 Department of Neuroscience Sheffield Institute for Translational Neuroscien University of Sheffield Sheffield United Kingdom; 13 Department of Psychology Beaumont Hospital Dublin Ireland; 14 Servicio de Neurología Hospital del Mar Barcelona Spain; 15 European Organization for Professionals and Patients with ALS (EUpALS) Leuven Belgium; 16 Department of Neurology Montreal Neurological Institute McGill University Montreal, QC Canada; 17 School of Biomedical Sciences Faculty of Medicine University of Queensland Brisbane Australia; 18 The Royal Brisbane and Women’s Hospital Herston Australia; 19 Wesley Medical Research the Wesley Hospital Auchenflower Australia; 20 Centre for Clinical Research University of Queensland Brisbane Australia; 21 Australian Institute for Bioengineering and Nanotechnology University of Queensland Brisbane Australia; 22 Advanced Digital Innovation (UK) Ltd Salts Mill United Kingdom; 23 Department of Health Evidence Section Biostatistics Radboud Medical Centre Nijmegen Nijmegen Netherlands; 24 Department of Neurology National Neuroscience Centre Beaumont Hospital Dublin Ireland; 25 FutureNeuro SFI Research Centre Royal College of Surgeons in Ireland Dublin Ireland

**Keywords:** amyotrophic lateral sclerosis, digital health care technology, e-health

## Abstract

Despite recent and potent technological advances, the real-world implementation of remote digital health technology in the care and monitoring of patients with motor neuron disease has not yet been realized. Digital health technology may increase the accessibility to and personalization of care, whereas remote biosensors could optimize the collection of vital clinical parameters, irrespective of patients’ ability to visit the clinic. To facilitate the wide-scale adoption of digital health care technology and to align current initiatives, we outline a road map that will identify clinically relevant digital parameters; mediate the development of benefit-to-burden criteria for innovative technology; and direct the validation, harmonization, and adoption of digital health care technology in real-world settings. We define two key end products of the road map: (1) a set of reliable digital parameters to capture data collected under free-living conditions that reflect patient-centric measures and facilitate clinical decision making and (2) an integrated, open-source system that provides personalized feedback to patients, health care providers, clinical researchers, and caregivers and is linked to a flexible and adaptable platform that integrates patient data in real time. Given the ever-changing care needs of patients and the relentless progression rate of motor neuron disease, the adoption of digital health care technology will significantly benefit the delivery of care and accelerate the development of effective treatments.

## Introduction

Remote digital health technology, ranging from simple mobile apps to implantable devices, will reform the delivery of care. Despite the recent technological advances in biosensors, mobile communications, and cloud computing, their real-world implementation remains to be fully realized [1]. Motor neuron disease (MND) is a debilitating disorder in which digital health care technology will benefit delivery of care [2,3] and also expedite the development of effective treatments [4-6]. Patients living with MND rapidly lose their functional independence, making travel, communication, and visits to specialist clinics for assessment of vital clinical parameters challenging [7]. This has been particularly the case during the COVID-19 pandemic [8,9]. The effects of the pandemic have focused attention on technological advances that increase the accessibility and personalization of care, and remote biosensors that optimize the collection of vital clinical parameters, irrespective of the patients’ ability to visit the clinic, are poised to revolutionize the clinical encounter. These developments hold benefits for both research and care settings, driving the implementation of real-world use of remote digital health care technology in MND [2,10].

Notwithstanding, the following key barriers delay the adoption and wide-scale implementation of digital health care technology in MND [2]: (1) technical complexities, (2) low compliance rates, (3) time investment required, (4) high costs, and (5) legislation challenges. For clinical trials, adoption is further complicated by regulatory hurdles because of reduced auditability, consistency, and data quality [6,11,12]. Although there is considerable work underway in these areas that will facilitate the validation and initial adoption of digital technology in MND [4,5,13-15], it is also the case that device or platform incompatibility might further delay their wide-scale adoption and large-scale data aggregation. Therefore, there is a recognized need to open collaborations among clinicians, engineers, and technology companies that will support early implementation. Here, we outline a road map that aims to (1) facilitate the identification of clinically relevant digital parameters; (2) mediate the development of benefit-to-burden criteria for innovative technology; and (3) direct the validation, harmonization, and adoption of digital health care technology in real-world settings.

## The Need for Remote Technology in MND

Disease heterogeneity and rapid physical deterioration are key drivers in the delivery of care and monitoring of disease progression in clinical trials. Although MND is recognized as a clinical entity, it is better considered as a number of different subgroups with variable clinical presentations, different causes, and different disease trajectories. A flexible approach toward management is required such that the patient’s clinical condition is monitored at intervals that best reflect both the needs of the patient and their family and the trajectory of the disease. As progression ultimately leads to severe disability, clinical attendance is increasingly burdensome [16], notwithstanding the known benefits of multidisciplinary clinics that include survival and optimal access to assistive devices or proven treatments [17]. Unfortunately, even in developed countries, attendance at multidisciplinary clinics is as low as 43%, and many of those who can attend are unable to return [18].

**Figure 1:**
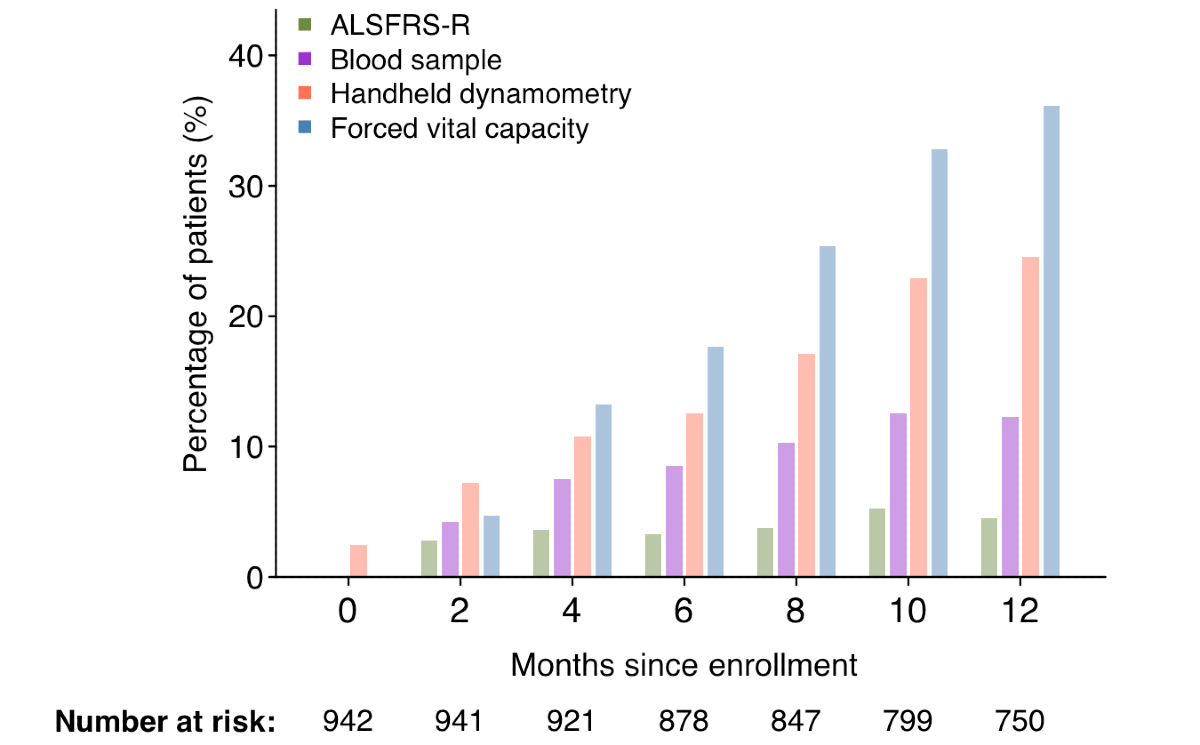
Percentage of EMPOWER patients with missing information on clinical outcome measures. For each clinical visit in the EMPOWER study, we determined the number of patients that were on study medication and were alive (ie, number at risk). Subsequently, we calculated the proportion of missing observations per outcome measure. If the patient was no longer able to visit the clinic (as indicated by the number of missing in-clinic muscle strength assessments), ALSFRS-R data and blood samples were collected remotely by home visit or by phone. ALSFRS-R: Amyotrophic Lateral Sclerosis Functional Rating Scale-Revised.

We illustrate the extent of this attrition process, as observed in a large clinical trial [19], in Figure 1. From the point of trial enrollment, almost 1 in 4 (184/750, 24.5%) patients failed to visit the clinic after 12 months, and more than one-third (271/750, 36.1%) were unable to provide reliable respiratory information. These figures are likely an underestimation of the attrition rates occurring at multidisciplinary clinics because clinical trials select for patients with better prognoses [20,21]. These high attrition rates affect the estimation of treatment benefit within clinical trials, reduce statistical power, and may lead to the continuation of an ineffective compound into a subsequent phase of clinical development.

Figure 1 also demonstrates that outcomes that can be measured remotely, either by phone for the Amyotrophic Lateral Sclerosis Functional Rating Scale-Revised (ALSFRS-R) or by a home visit for a blood sample, can reduce attrition and may mitigate many of the current challenges. Remote data collection can be tailored to identify and accommodate the ongoing needs of patients and caregivers, thus shortening the time spent at the hospital or traveling to the hospital and personalizing visiting schemes [14,22]. This in turn can provide a communication channel between patients and health care providers that builds an iterative and shared decision-making process regarding the timing and management of interventions and the approaches to end of life [23]. For clinical trials, home-based monitoring of disease progression is likely to more accurately reflect the patients’ true physical condition in free-living (ie, nonclinical) settings and lower the burden of monitoring. This in turn is likely to increase the number of patients who can participate in clinical research and provide real-world insight into the therapeutic value of experimental drugs [6,11,24]. Designing an information source that reflects the patients’ true physical condition in near real time is therefore of significant value for all stakeholders.

## A Road Map for MND

In this paper, we considered technology that assists with remote monitoring of MND disease progression or identifies critical health issues such as the development of respiratory or nutritional failure. To that end, we defined two key end products of the road map, as follows:

1. A set of reliable digital parameters to capture data, collected under *free-living* conditions at the patient’s home, that reflect patient-centric measures and facilitate clinical decision making
2. An integrated open-source system that provides personalized feedback to patients, health care providers, clinical researchers, and caregivers

To achieve this, there is a need for input from all stakeholders, including those living with MND and those who (informally) care for patients with MND. To promote the uptake of new technology in real-world settings, we used a user-centered co-design approach that involves input from stakeholders (eg, patients, caregivers, physicians, ethicists, regulators, programmers, and researchers) at every stage of development [25]. There will be specific attention paid to patient, caregiver, and researcher access as well as international regulatory, financial, and licensing hurdles to ensure widespread implementation, sustainability, and growth [26]. For this road map, we have identified the following three key questions: (1) what to measure; (2) how to measure; and (3) how to implement? We outlined the requirements to answer each of these questions, considering the needs of the various stakeholders of the technology.

## What to Measure?

Patient centricity is crucial to promote long-term adherence, where end points should focus on how aspects of MND affect daily living and distinguish between symptoms (ie, impairments) and activities (ie, disabilities). The clinician’s input may help to prioritize end points in clinically relevant domains or supplement other aspects of MND that patients may not recognize as clinically important (eg, weight loss or cognitive and behavioral changes). This approach maximizes the likelihood of acquiring data that facilitate clinically relevant decision making and minimizes the collection of redundant information.

### Workplan: What to Measure?

**Figure 2:**
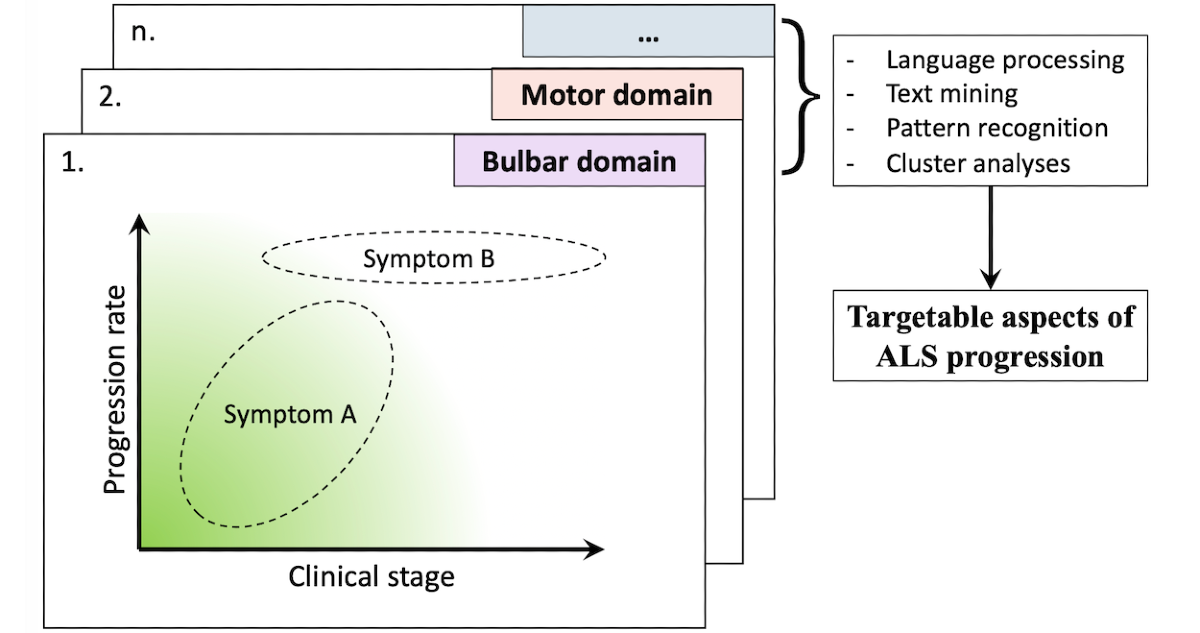
Untangling clinical heterogeneity in motor neuron disease in a 3D framework. The prevalence of symptoms as reported by patients will be mapped according to their clinical stage and (predicted) progression rate. Clusters of symptoms will be defined into domains (eg, bulbar, motor, respiratory, and cognition) for creating a 3D map of motor neuron disease symptom heterogeneity. The map will be used to identify clinically relevant and targetable end points. ALS: amyotrophic lateral sclerosis.

Information will be collected from three independent sources: (1) a systematic literature review summarizing and meta-analyzing all published symptomology associated with MND; (2) face-to-face in-depth interviews and focus group discussions with patients, caregivers, and physicians until data saturation is reached; and (3) a cross-sectional, population-based patient survey. We will apply a purposive and theoretical sampling strategy to ensure that we capture the complete range of disease patterns reported among patients with MND [27]. Videoconferencing will be used to ensure full patient participation at all meetings. In Figure 2, we illustrate two key elements that may help to disentangle the clinical heterogeneity observed among patients and guide sampling strategies or evaluate generalizability: clinical disease stage and progression rate. Some symptoms such as speech disturbances may only be prevalent in early clinical stages and will disappear over time owing to a complete loss of function (illustrated as *Symptom A*). Conversely, some symptoms may only be prevalent in patients with a certain disease type or progression rate (eg, symptoms related to spasticity in a primarily upper motor neuron, a slowly progressing subtype of disease, illustrated as *Symptom B*). Mapping the prevalence of symptoms in this 2D plane may help to disentangle a large part of the clinical heterogeneity in MND. Clinical stage can be defined by the King’s clinical and Milano-Torino functional staging algorithms [28,29], whereas the progression rate is directly related to survival time and could be mapped by, for example, the patient’s predicted prognosis [30].

A complete list of symptoms will be extracted from the interviews and focus group meetings, supplemented with data from the literature review, and by language processing and text mining from a population-based, cross-sectional survey asking open questions such as “What bothers you the most about your MND” and “In what way does this affect your daily living?” Information collection will be conducted in a multicenter, international setting to account for potential geographical, social, and cultural differences. Finally, a large range of statistical methods will be used for pattern recognition to cluster targets and end points, identify redundant information, and classify subgroups of patients with specific needs. Ultimately, this results in a 3D framework, as depicted in Figure 2, that orders a large part of the variability in symptoms among patients. Additional classification using, for example, cognitive or behavioral impairment or the presence of dementia may be applied to further elucidate heterogeneity in MND [31,32]. The 3D framework could help to evaluate the representativeness of the included population and quickly identify clinically relevant and targetable end points. The final list of targetable end points will be evaluated by patients, caregivers, and physicians for further refinement and prioritization.

## How to Measure?

The second step is to translate the list of clinically relevant aspects of MND into digital parameters. For example, speech is a clinically relevant outcome in which a mobile speech app could serve to improve its objective assessment in a standardized manner. Critical aspects to consider here are, among other things, whether the mobile app requires an action-dependent or passive task (eg, repeat a fixed sentence vs record a daily 5-min conversation), the uniformity and precision of the digital voice parameters (eg, speaking rate vs volume vs perturbations) [33], the required number and length of measurements (eg, daily, weekly, or monthly follow-up), and whether direct supervision or caregiver assistance is required. These factors not only determine the burden for patients or caregivers but also establish the value of the obtained information for researchers and health care providers. The aim is to define selection criteria for digital technology that evaluates burden-to-information ratios and, based on these criteria, results in a standardized and protocolized set of digital metrics for MND.

### Workplan: How to Measure?

We will conduct a series of systematic reviews to define a list of candidate technologies for each targetable aspect of MND. Naturally, the real-world use of the suggested technology could reveal insights that may not have been reported previously in the literature or by the supplier. Longitudinal studies are preferred because they not only evaluate test-retest reliability and the validity to capture disease progression of a digital parameter but they also provide information on patient adherence and the adequacy of the monitoring protocol (eg, obtaining daily, weekly, or monthly measurements). The latter is an often-overlooked aspect, although it has important consequences for all stakeholders. Naturally, a daily monitoring scheme would provide the most information about the disease for health care providers and researchers [5] but could disproportionally increase patient burden and lead to high (differential) attrition. A periodic monitoring scheme may be more successful, and a data-driven optimization of this trade-off, while accounting for patient and health care provider preferences, should be used.

As a practical illustration, in Table 1, we show the benefit of changing the monitoring frequency from monthly to daily for two end points: ALSFRS-R and daily physical activity [4]. The benefit of more or less frequent monitoring strongly depends on the end point, and as demonstrated by the ALSFRS-R, it could be questionable whether high-frequency monitoring is always beneficial. In addition, because of a difference in progression rates among patients, the monitoring frequency could be further optimized for each individual patient (eg, a patient who slowly progresses may require less frequent monitoring). Ultimately, the optimal monitoring scheme is likely to differ from digital end point to end point, and longitudinal data are essential to estimate a data-driven optimal burden-to-information ratio for each device or mobile app [4].

**Table 1:** Sample size estimates for the Amyotrophic Lateral Sclerosis Functional Rating Scale-Revised (ALSFRS-R) and daily activity with varying monitoring frequencies.^a^

Characteristic			ALSFRS-R		Daily activity
Monthly progression rate^b^			−0.06		−0.05
Between-patient variability, σ^2^_between_			0.06^2^		0.04^2^
Within-patient variability, σ^2^_within_			0.15^2^		0.54^2^
**Required sample size (6-month follow-up), n (% difference)**					
	Monthly	226 (ref^c^)		1208 (ref)	
	Biweekly	210 (−7)		805 (−33)	
	Weekly	199 (−12)		522 (−57)	
	Daily	187 (−17)		214 (−82)	
**Required sample size (12-month follow-up), n (% difference)**					
	Monthly	191 (ref)		322 (ref)	
	Biweekly	189 (−1)		251 (−22)	
	Weekly	187 (−2)		209 (−35)	
	Daily	185 (−3)		168 (−48)	

^a^Daily activity was defined as the proportion of time that the patient was nonsedentary, as described elsewhere [4]. Sample size calculations are based on a standardized linear mixed model; data are based on 42 patients [4]. Sample size calculations assumed a target power of 80%, 2-sided α of 5%, and a 25% reduction in the progression rate; numbers are the required sample size per group [34].

^b^Progression rate is expressed as number of SD per month. Both the ALSFRS-R and daily activity were standardized to make a direct comparison possible. The ALSFRS-R has, on average, a faster progression rate, more variability between patients, and less variability within patients compared with daily activity.

^c^ref: reference.

There are circumstances in which digital technology may not appropriately quantify disease symptoms (eg, changes in mood or alterations in cognition). Therefore, an important aspect of the road map is to additionally develop scales and questionnaires that can be administered remotely to quantify clinically relevant, subjective symptoms [35]. It is important for these patient- and caregiver-reported outcome measures to consider elements such as the ease of engagement (eg, mobile apps with multiple verbal and nonverbal response formats), the length of questionnaires, the validity of questions (eg, differences between patient-reported and in-clinic outcomes), and the viability for digitization. Ultimately, it may prove critical to harmonize and integrate passive digital methods such as accelerometry with task-dependent methods and questionnaires to derive the most reliable and representative state of disease at any given time.

In addition, an important goal is to maximize the adherence and retention of the patient or caregiver. In two studies that reported on remote monitoring with personalized feedback in care settings [14,22], 80%-87% of the patients provided regular ALSFRS-R information. In two recent studies that solely monitored disease progression without providing care, this was reduced to 24%-56% [15,36]. It seems critical to provide an incentive to participate, where patients do not simply upload their data into the cloud but also receive something in return, such as care, tailored information, or personalized insights. An understanding of how best to retain patients in the digital environment (eg, by incorporating behavioral or motivational techniques) and which patient factors are related to digital uptake or adherence are fundamental to the success of the workplan. Finally, it is important to consider the geographical region and the local facilities and demographics. Older populations or patients from developing areas with minimal financial, technological, or educational facilities may encounter challenges when using devices. This could increase the risk of care asymmetries across populations and should be considered when comparing candidate technologies.

## How to Implement?

A standardized format or platform for multiple digital metrics is not yet available, and a priority of the Treatment Research Initiative to Cure ALS will be to provide this technology [37]. This platform will solve the problem of collecting data relating to multiple domains while using multiple apps from different providers. Such a platform is essential because clinical decision making is often multidimensional and requires insight into various aspects of the patient simultaneously. The aim of this final part of the road map is to (1) integrate different data sources required by the end users and (2) deploy an open-source framework product in which one can flexibly integrate different third-party solutions, evaluate the performance of digital metrics compared with clinically relevant events, and allow in-depth analysis of health care expenditures and outcomes.

### Workplan: How to Implement?

**Figure 3:**
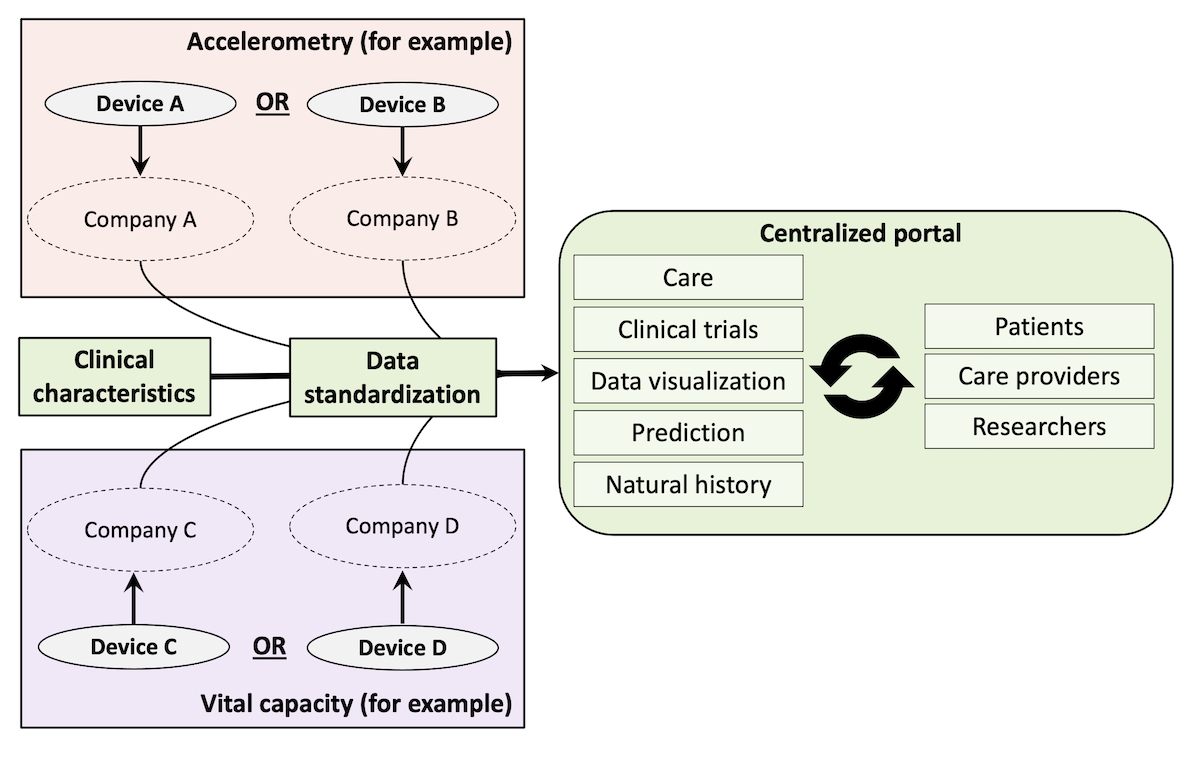
The harmonization and centralization of digital outcome data before dissemination. A schematic illustration of the integration of different devices from different third parties into a central database that acts as a single integrative platform is illustrated for vital capacity and accelerometry. Information and insights can subsequently be disseminated interactively to the respective end users.

In Figure 3, we schematically illustrate the final product. The first step would be to standardize the metrics that are of interest across different devices. The standardized metrics are subsequently stored in a central database that acts as a single integrative platform. The capability of the device to allow uninterrupted, remote data access is therefore critical; the final selection of devices or mobile apps is likely an iterative process in close relationship with the *How to Measure* section. Although the concept is simple, this part of the road map may be the most challenging because of, among other things, privacy, regulatory, ethical, ownership, licensing, certification, and database challenges. A close collaboration among various industry, academic, funding, and regulatory parties has been initiated to provide a clear central and local governance and management structure with meticulous planning.

The next step is to translate the centralized data into useful information and disseminate insights to the relative end user. Given the extent of information acquired and the number of different stakeholders and settings, a customized approach will be required. Where a trialist may be primarily interested in a historical benchmark of group averages, clinicians and patients will be principally interested in individual disease patterns. These interests need to be carefully documented, and a dynamic, user-centered co-design approach is vital. It is important to evaluate how the implementation of digital parameters affects existing standard operating procedures and work practices or how health care professionals adhere to new MND technologies [38]. For care purposes, defining optimal *activation* or *flagging* rules is important for timely activation of additional care. For example, thresholds are needed for respiratory function (or decline in function) to alert the patient’s health care provider or automatically refer the patient to a pulmonologist when the threshold is reached. Defining these rules can help to standardize remote monitoring of MND, and this requires the development of a disease-specific protocol based on MND guidelines and expert opinions [14].

In addition, efforts are required to evaluate different strategies for effective communication among users, for example, the implementation of chat functions between patients and health care providers or the provision of personalized information or feedback on the obtained measurements. By making use of open-source software platforms, tailored programs can be developed in a dynamic interface, while seamlessly integrating the continuously updating information from wearables and mobile apps. We have previously provided an example of such a platform for clinical trial design [39]. The platform and interactive platform will go through several stages before the product can be finalized, for example, prototype development, iterative testing phases, experimenting with varying key parameters (eg, user burden, monitoring frequency, different wording and timings, and different prompting regimes), pilot releases, and upgrading, taking into account the input from all users at every stage.

Finally, only by real-world implementation can the true value of digital parameters and platforms be evaluated. For care settings, it is important to conduct dedicated studies to evaluate the cost-effectiveness of the digital health care technology and its effect on clinical outcomes. The gold standard would be randomized controlled trials, for example, allocating patients to usual care or digital assisted care or allocating hospitals in a cluster randomized study. An example of the former has been published previously, in which the authors evaluated the effect of home telemonitoring of noninvasive ventilation on the number of hospital visits [40], together with its cost-effectiveness [41]. Given the complexity of most eHealth interventions [13], such trials are challenging and it may not be immediately clear what an ideal efficacy end point would be (eg, survival time, number of hospital admissions, costs, or patient-reported indicators?). A key aspect for successful uptake is to have a financial model that is sustainable, while providing clinically relevant benefits to all stakeholders.

For clinical trials, the cost aspect may be of similar importance, and studies evaluating the gain in retention compared with cost would be highly insightful. Besides the potential reduction in attrition rates and gain in information quality, it will be necessary to determine whether digital metrics can ultimately replace the clinical efficacy end point (ie, surrogacy) and increase trial efficiency. The real-world systematic use of digital health care technology, alongside common clinical efficacy endpoints, is therefore a key first step. The potential use of real-world data originating from digital health care technology in clinical drug development has been recognized for regulatory purposes [42]. Nevertheless, important challenges remain, such as the design of interventional studies and challenges related to data availability, data quality, auditability, and the completeness of electronic phenotyping [43].

## Data Security and Privacy Regulations

Given the current data security and privacy regulations for medical devices and personal data (eg, General Data Protection Regulation), data security and ethical hurdles will have a major impact on the developmental and operational aspects of the platform and on how the data may be used during (international) research projects or within care settings. These aspects are not only important for the platform itself but also apply to, for example, cloud services from third parties. How, when, and by whom data can be accessed need to be tailored and defined for each participating site individually to comply with local laws and regulations. Making use of existing infrastructures, for example, by integrating the platform into eHealth care services and requiring two-factor authentication could facilitate secure data access [14], but its feasibility depends on the available local facilities. In addition, considerations for using the data include obtaining consent to (1) share personally identifiable information for direct care only, (2) share personally identifiable information for research and care purposes, or (3) share anonymized data to be used for research only. Similarly, there is a need to address challenges in intellectual property and data ownership (eg, where the demarcation might be made between clinical and technical intellectual property).

An important consideration is the CE (Conformité Européenne) marking for medical devices and software. The disadvantage of CE marking an entire ecosystem, as presented in Figure 3, is that it may significantly constrain the rate of change and evolution of the system. CE marking locks down the design, and any significant changes made to the system (eg, addition of new algorithms or devices) requires reapproval by external regulatory bodies. A consideration could be to use a mixed approach of using CE-marked devices and algorithms interconnected and facilitated by non–CE-marked software. This will require some design constraints to make it possible (eg, there can be no opacity in terms of non–CE-marked algorithms supporting clinical decision making), and achieving this goal will require careful design and partitioning from the outset. Another strategy is to consolidate and rationalize into a single CE-marked system once the ecosystem for ALS, including measurements, algorithms, and protocols, reaches maturity. These challenges will require dedicated strategies, which will need to be developed with the involvement of all relevant stakeholders and by obtaining expertise from ethicists, regulators, and data privacy officers. Ultimately, these discussions may affect the choice of measurement or device, indicating that the final set of digital parameters for MND is likely defined during a dynamic and iterative process rather than being fixed from the outset.

## Conclusions

In conclusion, this road map aims to align the current developments in digital health care technology for MND and initiate a collaborative effort to mediate its wide-scale adoption across MND clinics and clinical trials. We defined the following three key questions, the answers to which are critical to achieve this aim: (1) what to measure; (2) how to measure; and (3) how to implement? Each question requires a dedicated study methodology to overcome potential implementation, adoption, or regulatory hurdles, which are summarized in Table 2.

**Table 2:** Summary of the road map for remote digital health technology for motor neuron disease (MND).

Question	Aim	Method	Result
What to measure?	To define clinically relevant, patient-centric, and targetable aspects of MND	Inventorying symptomology associated with MND by systematic literature review, face-to-face in-depth interviews, focus group discussions, and population-based patient surveys	List of targetable end points for MND
How to measure?	To translate targetable end points for MND into digital parameters	Candidate technologies are identified by systemic literature reviews. Standardized longitudinal studies are initiated to evaluate test-retest reliability and validity to capture disease progression and to assess protocol adherence.	Set of standardized and protocolized digital metrics
How to implement?	To centralize digital metrics in an integrative platform and disseminate user-dependent information	Create a single database using open-source software platforms, reactive programming, and third-party cloud services Initiation of dedicated studies to evaluate health care expenditures, cost-effectiveness outcomes, and surrogate biomarker value	Scalable and validated platform to provide tailored information for care and research settings

Given the ever-changing care needs of patients and the relentless progression rate of MND, aligning our current endeavors and facilitating the adoption of digital health care technology will significantly benefit the delivery of care and accelerate the development of effective treatments against this debilitating disease.

## Abbreviations

ALS: amyotrophic lateral sclerosis
ALSFRS-R: Amyotrophic Lateral Sclerosis Functional Rating Scale-Revised
MND: motor neuron disease
CE: Conformité Européenne
